# Heralded high-efficiency quantum repeater with atomic ensembles assisted by faithful single-photon transmission

**DOI:** 10.1038/srep15610

**Published:** 2015-10-27

**Authors:** Tao Li, Fu-Guo Deng

**Affiliations:** 1 Department of Physics, Applied Optics Beijing Area Major Laboratory, Beijing Normal University, Beijing 100875, China; 2 State Key Laboratory of Networking and Switching Technology, Beijing University of Posts and Telecommunications, Beijing 100876, China

## Abstract

Quantum repeater is one of the important building blocks for long distance quantum communication network. The previous quantum repeaters based on atomic ensembles and linear optical elements can only be performed with a maximal success probability of 1/2 during the entanglement creation and entanglement swapping procedures. Meanwhile, the polarization noise during the entanglement distribution process is harmful to the entangled channel created. Here we introduce a general interface between a polarized photon and an atomic ensemble trapped in a single-sided optical cavity, and with which we propose a high-efficiency quantum repeater protocol in which the robust entanglement distribution is accomplished by the stable spatial-temporal entanglement and it can in principle create the deterministic entanglement between neighboring atomic ensembles in a heralded way as a result of cavity quantum electrodynamics. Meanwhile, the simplified parity-check gate makes the entanglement swapping be completed with unity efficiency, other than 1/2 with linear optics. We detail the performance of our protocol with current experimental parameters and show its robustness to the imperfections, i.e., detuning and coupling variation, involved in the reflection process. These good features make it a useful building block in long distance quantum communication.

Quantum mechanics provides some interesting ways for communicating information securely between remote parties^1^,^2^,^3^,^4^,^5^. However, in practice the quantum channels such as optical fibers are noisy and lossy^6^. The transmission loss and the decoherence of photon systems increase exponentially with the distance, which makes it extremely hard to perform a long-distance quantum communication directly. To overcome this limitation, Briegel *et al.*^7^ proposed a noise-tolerant quantum repeater protocol in 1998. The channel between the two remote parties *A* and *B* is divided into smaller segments by several nodes, the neighboring nodes can be entangled efficiently by the indirect interaction through flying qubits, and the entanglement between non-neighboring nodes is implemented by quantum entanglement swapping, which can be cascaded to create the entanglement between the terminate nodes *A* and *B*.

The implementation of quantum repeaters is compatible with different physical setups assisted by cavity quantum electrodynamics, such as nitrogen vacancy centers in diamonds^8^, spins in quantum dots^9^,^10^,^11^,^12^, single trapped ions or atoms^13^,^14^. However, the most widely known approach for quantum repeaters is based on atomic ensembles^15^ due to the collective enhancement effect^16^. In a seminal paper by Duan *et al.*^17^, the atomic ensemble is utilized to act as a local memory node. The heralded collective spin-wave entanglement between the neighboring nodes is established by the detection of a single Stokes photon, emitted indistinguishably from either of the two memory nodes via a Raman scattering process. However, due to the low probability of Stokes photon emission required in the Duan-Lukin-Cirac-Zoller (DLCZ) proposal^17^, the parties can hardly establish the entanglement efficiently for quantum entanglement swapping. In order to improve the success probability, photon-pair sources and multimode memories are used to construct a temporal multi-mode modification^18^, and then the schemes based on the single-photon sources^19^ and spatial multiple modes^20^ are developed. Besides these protocols based on Mach-Zehnder-type interference, Zhao *et al.*^21^,^22^ proposed a robust quantum repeater protocol based on two-photon Hong-Ou-Mandel-type interference, which relaxes the long-distance stability requirements and suppresses the vacuum component to a constant item. Subsequently, the single-photon sources are embedded to improve the performance of robust quantum repeaters^23^,^24^,^25^. In addition, Rydberg blockade effect^26^ is used to perform controlled-NOT gate between the two atomic ensembles in the middle node^27^,^28^, which makes the quantum entanglement swapping operation be performed deterministically.

Since the two-photon interference is performed with the polarization degree of freedom (DOF) of the photons^21^,^22^, which is incident to be influenced by the thermal fluctuation, vibration, and the imperfection of the fiber^29^, the fidelity of the entanglement created between the neighboring nodes will be decreased when the photons are transmitted directly^6^,^7^. In other words, the more the overlap of the initial photon state used in the two-photon interference is, the higher the fidelity of the entanglement created is. Following the idea of Zhao’s protocol^21^, quantum repeaters immune to the rotational polarization noise are proposed with the time-bin photonic state^30^ and the antisymmetric Bell state^31^ 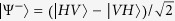, respectively. When the noise on the two orthogonal polarized photon states is independent, Zhang *et al.*^32^ utilized the faithful transmission of polarization photons^29^ to surmount the collective noise. In the ideal case, the two-fold coincidence detection in the central node can successfully get the stationary qubits entangled maximally in a heralded way. Apart from this type of entanglement distribution, Kalamidas^33^ proposed an error-free entanglement distribution protocol in the linear optical repeater. An entangled photon source is placed at the center node, and the entangled photons transmitted to neighboring nodes are encoded with their time-bin DOF. With two fast Pockels cells (PCs), the entanglement distribution can be performed with a high efficiency when the polarization-flip-error noise is relatively small.

In a recent work, Mei *et al.*^34^ built a controlled-phase-flip (CPF) gate between a flying photon and an atomic ensemble embedded in an optical cavity, and constructed a quantum repeater protocol, following some ideas in the original DLCZ scheme^17^. In 2012, Brion *et al.*^35^ constituted a quantum repeater protocol with Rydberg blocked atomic ensembles in fiber-coupled cavities via collective laser manipulations of the ensembles and photon transmission. Besides, Wang *et al.*^36^ proposed a one-step hyperentanglement distillation and amplification proposal, and Zhou and Sheng^37^ designed a recyclable protocol for the single-photon entanglement amplification, which are quite useful to the high dimensional or multiple DOFs optical quantum repeater.

In this paper, we give a general interface between a polarized photon and an atomic ensemble trapped in a single-sided optical cavity. Besides, we show that a deterministic faithful entanglement distribution in a quantum repeater can be implemented with the time-bin photonic state when two identical fibers act as the channels of different spatial DOFs of the photons. Interestingly, it does not require fast PCs and the time-slot discriminator^29^,^30^,^31^,^32^,^33^ is not needed anymore. By using the input-output process of a single photon based on our general interface, the entanglement between the neighboring atom ensembles can be created in a heralded way, without any classical communication after the clicks of the photon detectors, and the quantum swapping can be implemented with almost unitary success probability by a simplified parity-check gate (PCG) between two ensembles, other than 1/2 with linear optics. We analyze the performance of our high-efficiency quantum repeater protocol with current experimental parameters and show its robustness to the imperfections involved in the reflection process. These good features will make it a useful building block in long-distance quantum communication in future.

## Results

### A general interface between a polarized photon and an atomic ensemble

**Figure 1:**
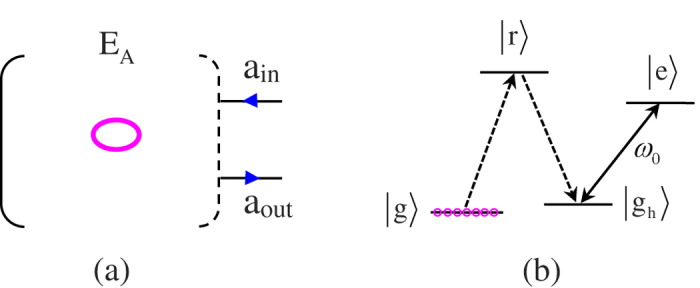
(**a**) Schematic diagram for a single-sided cavity coupled to an atomic ensemble system. (**b**) Atomic level structure.

The elementary node in our quantum repeater protocol includes an ensemble with *N* cold atoms trapped in a single-sided optical cavity^34^,^35^. The atom has a four-level internal structure and its relevant levels are shown in Fig. 1. The two hyperfine ground states are denoted as 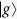 and 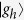. The excited state 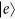 and the Rydberg state 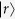 are two auxiliary states. The 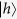 polarized cavity mode *a*_*h*_ couples to the transition between 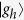 and 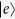. Initially, all of the atoms are pumped to the state 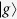. With the help of the Rydberg state 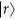, one can efficiently perform an arbitrary operation between the ground state 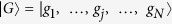 and the single collective spin-wave excitation state^17^ 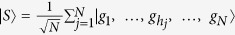 via collective laser manipulations of the ensembles^34^,^35^,^38^. The single collective excited state 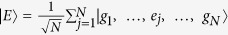. When the Rydberg blockade shift is of the scale 2*π* × 100 *MHz*, the transition between 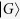 and 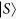 can be completed with an effective coupling strength 2*π* × 1 *MHz* and the probability of nonexcited and doubly excited errors^39^ is about 10^−3^–10^−4^. Recently, rotations along axes *R*_*x*_, *R*_*y*_, and *R*_*z*_ of a spin-wave excitation with an average fidelity of 99% are achieved in ^87^*Rb* atomic ensembles and they are implemented by making use of stimulated Raman transition and controlled Larmor procession^40^. In other words, the high-efficiency single qubit rotations of the atomic ensemble can be implemented faithfully.

Let us consider an 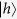 polarized input photon with the frequency *ω*, which is nearly resonant to the cavity mode 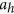 with the frequency *ω*_*c*_. The coupling rate between the cavity and the input photon can be taken to be a real constant 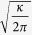 when the detuning |*δ*′| = |*ω* − *ω*_*c*_| is far less than the cavity decay rate *κ* (|*δ*′| ≪ *κ*)^41^,^42^,^43^. The Hamiltonian of the whole system, in the frame rotating with respect to the cavity frequency *ω*_*c*_, is (*ħ* = 1)^41^


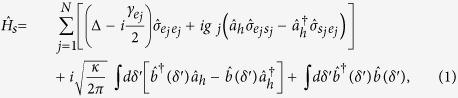


where 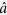 and 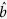 are the operators of the cavity mode and the input photon with the properties 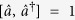 and 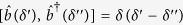, respectively. Δ = *ω*_0_ − *ω*_*c*_ is the detuning between the cavity mode frequency *ω*_*c*_ and the dipole transition frequency *ω*_0_, 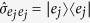, and 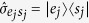. 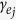 represents the spontaneous emission rate of the excited state 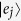, while *g*_*j*_ denotes the coupling strength between the *j-th* atom transition and the cavity mode 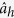. Here and after, we assume *g*_*j*_ = *g* and 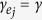 for simplicity.

With the Hamiltonian 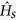 shown in Eq. (1), the Heisenberg-Langevin equations of motion for cavity 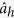 and the atomic operator 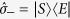 taking into account the atomic excited state decay *γ* can be detailed as^41^


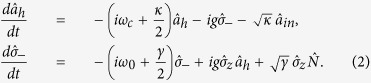


Here the Pauli operator 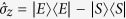, while 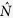 is corresponding to the vacuum noise field that helps to preserve the desired commutation relations for the atomic operator. Along with the standard cavity input-output relation 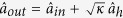, one can obtain the reflection and noise coefficients *r*(*δ*′) and *n*(*δ*′) in the weak excitation approximation where the ensemble is hardly in the state 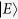 but predominantly in 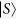, that is,


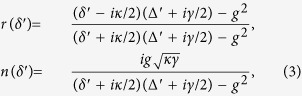


where Δ′ = *ω* − *ω*_0_ represents the frequency detuning between the input photon and the dipole transition. |*r*(*δ*′)|^2^ + |*n*(*δ*′)|^2^ = 1 means that when the noise field is considered, the energy is conserved during the input-output process of the single-sided cavity.

If the atomic ensemble in the cavity is initialized to be the state 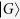, it does not interact with the cavity mode (i.e., *g* = 0). The input 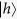 polarized probe photon feels an empty cavity and will be reflected by the cavity directly. Now, the reflection coefficient can be simplified to be^41^


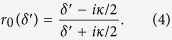


**Figure 2:**
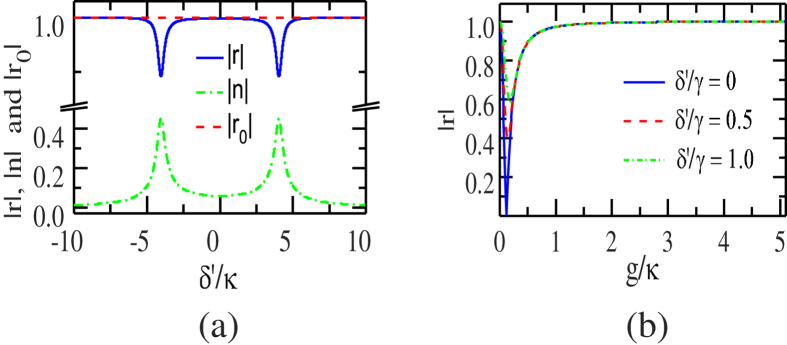
(**a**) |*r*| |*n*| and |*r*_0_| *vs* the scaled detuning *δ*′/*κ*, with the scaled coupling rate^53^ *g*/*κ* = 4.0566 and *γ*/*κ* = 0.0566. (**b**) |*r*| *vs* the scaled coupling rate *g*/*κ* with detuning *δ*′/*γ* = 0, 0.5, and 1.

**Figure 3:**
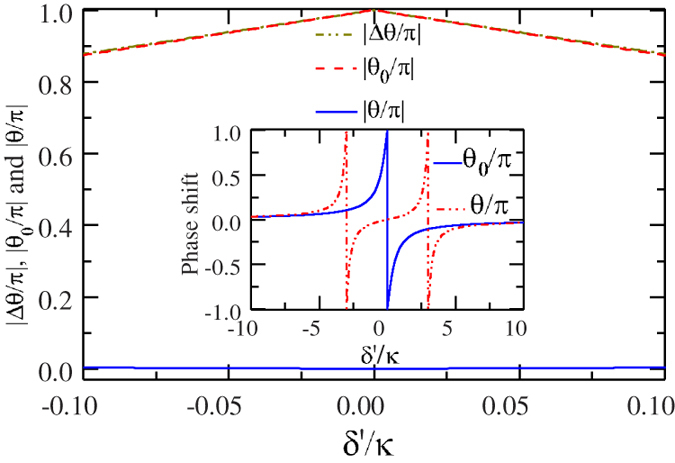
The absolute phase shifts *vs* the scaled detuning. The dashed and dashed-dot lines show the absolute phase shifts |*θ*_0_/*π*| and |*θ*/*π*| that the reflected photon gets, with the ensemble in the states 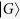 and 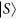, respectively. The solid line represents the absolute value of the phase shifts difference |Δ*θ*/*π*| = |*θ*_0_/*π* − *θ*/*π*|. The inset shows the phase shifts *vs* the scaled detuning. *θ*_0_/*π* and *θ*/*π* that the reflected photon gets, with the ensemble in 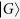 and 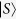, respectively.

Note that the detuning is small 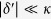, the pulse bandwidth is much less than the cavity decay rate *κ*. If the strong coupling condition 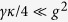 is achieved, one can get the input probe photon totally reflected with 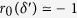 or 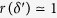, shown in Fig. 2. The absolute phase shifts versus the scaled detuning are shown in Fig. 3.

### Hybrid CPF gate on a photon-atomic-ensemble system and PCG on a two-atomic-ensemble system

**Figure 4:**
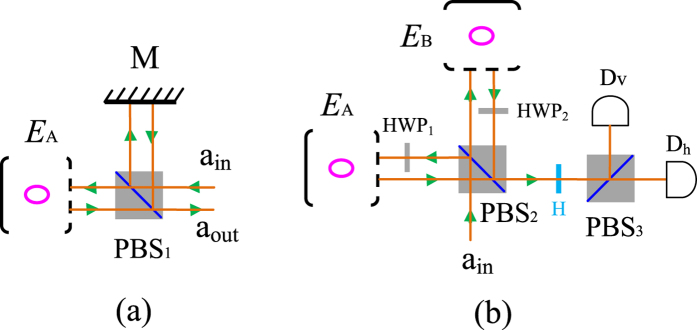
Schematic setup for implementing a CPF gate and a PCG. *M* stands for a mirror and the PBS transmits the 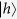 polarized photon and reflects the 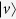 component. HWP_1_ and HWP_2_ are half-wave plates performing the bit-flip operation, while H represent a Hadamard rotation.

The principle of our CPF gate on a hybrid quantum system composed of a photon *p* and an atomic ensemble *E*_*A*_ is shown in Fig. 4, following some ideas in previous works^34^,^42^,^43^. Suppose that the photon *p* is in the state 
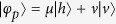
 (|*μ*|^2^ + |*ν*|^2^ = 1) and the ensemble *E*_*A*_ is in the state 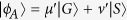 (|*μ*′|^2^ + |*ν*′|^2^ = 1). The 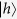 polarized component of the photon *p* transmits the polarization beam splitter (PBS) and then be reflected by the cavity, while the 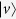 polarized component is reflected by the mirror *M*. The optical pathes of the 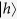 and 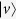 components are adjusted to be equal and they will be combined again at the PBS with an extra *π* phase shift on the 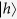 component if the ensemble is in the state 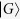. This process can be described as


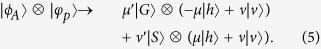


That is to say, the setup in Fig. 4(a) can be used to accomplish a CPF gate on the atomic ensemble *E*_*A*_ and the photon *p*.

The schematic diagram of our PCG on two atomic ensembles *E*_*A*_ and 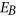 is shown in Fig. 4(b). Let us assume that *E*_*A*_ and *E*_*B*_ are initially in the states 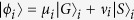 (|*μ*_*i*_|^2^ + |*ν*_*i*_|^2^ = 1 and *i* = *A*, *B*). One can input a polarized photon *p* in the state 
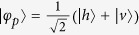
 into the import of the setup. HWP_1_ (HWP_2_) is used to perform the bit-flip operation 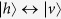 on the photon *p* by using a half-wave plate (HWP) with its axis at *π*/4 with respect to the horizontal direction. After the two components of *p* are reflected by the two cavities, they combine with each other at PBS_2_. The state of the system composed of the two atom ensembles and the photon evolves to be


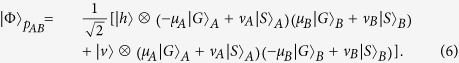


And then, another HWP names *H* whose axis is placed at *π*/8 is used to perform a Hadamard rotations 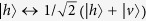 and 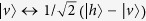 on the photon. The state of the system becomes


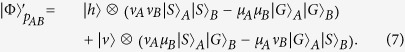


After the photon is measured with PBS_3_ and two single-photon detectors, the parity of *E*_*A*_ and *E*_*B*_ can be determined. In detail, if the photon is in the state 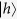, the two ensembles *E*_*A*_ and *E*_*B*_ have an even parity. If the photon is in 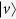, *E*_*A*_ and *E*_*B*_ have an odd parity. With an effective input-output process of a single photon, one can efficiently complete the PCG on two atomic ensembles.

### Entanglement distribution with faithful single-photon transmission

**Figure 5:**
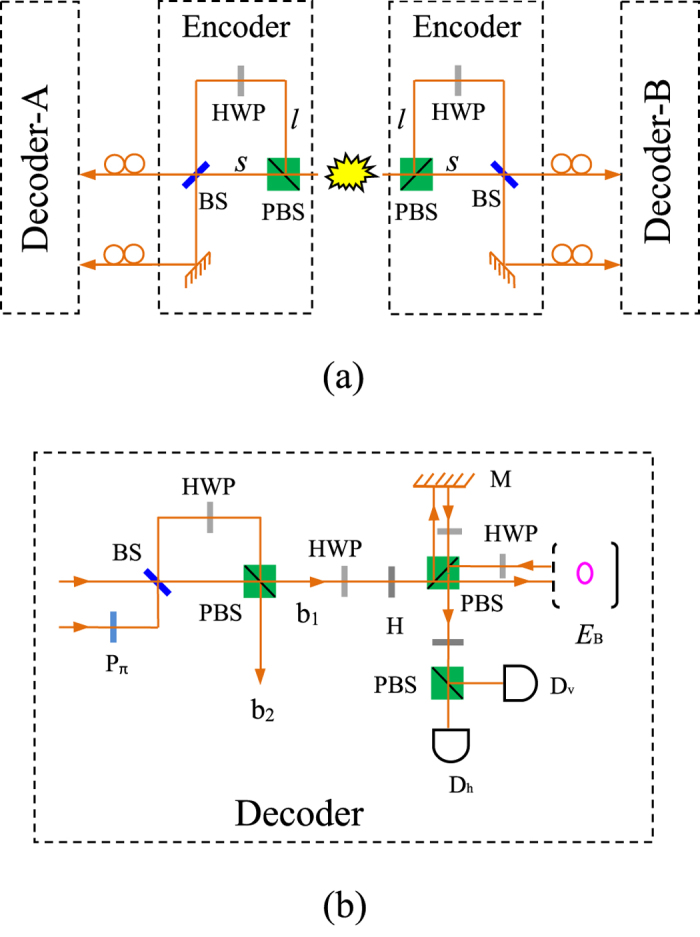
Schematic setup for entanglement distribution. Here p_*π*_ is a *π* phase shifter.

Suppose that there is an entanglement source which is placed at a central station between two neighboring nodes, say Alice and Bob. The source produces a two-photon polarization-entangled Bell state 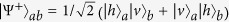. Here the subscripts *a* and *b* denote the photons sent to Alice and Bob, respectively. As shown in Fig. 5(a), the photons *a* and *b* will pass through an encoder in each side before they enter the noisy channels. The encoder is made up of a PBS, an HWP, and a beam splitter (BS). Here BS is used for a Hadamard rotation on the spatial DOF of the photon, i.e., 
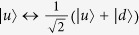
 and 
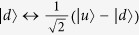
, where 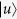 and 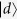 represent the upper and the down ports of the BS, respectively.

With our faithful single-photon transmission method (see Method), Alice and Bob can share photon pairs in a maximally entangled state, shown in Fig. 5. In detail, after a photon pair from the source passes through the two encoders, its state becomes


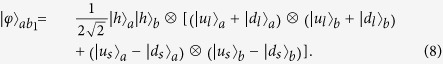


As the two photons *a* and *b* suffer from independent collective noises from the two channels, the influence of the channels on the two photons can be described with two unitary rotations 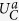 and 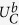 as follows:


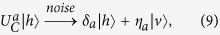



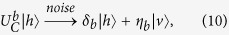


where |*δ*_*i*_|^2^ + |*η*_*i*_|^2^ = 1 (*i* = *a*, *b*). The influence on the polarization of the photons arising from the channel noises can be totally converted into that on the spatial DOF. The state of the photons *a* and *b* arriving at Alice and Bob becomes


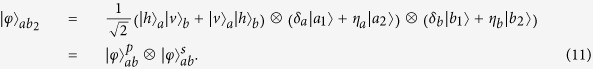


This is a two-photon Bell state 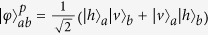 in the polarization DOF of the photon pair *ab*. Simultaneously, it is a separable superposition state 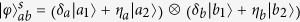 in the spatial DOF.

To entangle the stationary atomic ensembles *E*_*A*_ and *E*_*B*_, which are initialized to be 
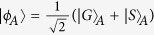
 and 
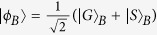
, only two CPF gates are required if Alice and Bob have shared some photon pairs in the Bell state 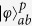. Let us take the case that the photons *a* and *b* come from the spatial modes *a*_2_ and *b*_2_ as an example to detail the entanglement creation process. As for the other cases, the same entanglement between *E*_*A*_ and *E*_*B*_ can be obtained by a similar procedure with or without some single-qubit operations.

First, the photon *a* suffers a Hadamard operation by passing through a half-wave plate *H*. Second, it is reflected by the cavity or the mirror *M*, which is used to complete the CPF gate on the photon *a* and the ensemble *E*_*A*_. Third, Alice performs another Hadamard operation on the photon *a*. Now, the state of the composite system composed of the photons *a* and *b* and the ensembles *E*_*A*_ and *E*_*B*_ evolves into 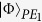,


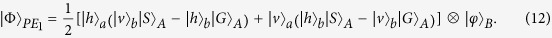


Fourth, Alice measures the polarization state of the photon *a* with a setup composed of PBS and single-photon detectors *D*_*h*_ and *D*_*v*_. If an 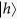 polarized photon is detected, the hybrid system composed of *b*, *E*_*A*_, and *E*_*B*_ will be projected into


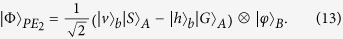


If a 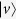 polarized photon is detected, the remaining hybrid system can also be transformed into the state 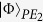 by a bit-flip operation 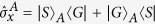 on the ensemble *E*_*A*_.

Up to now, the original entanglement of the photon pair *ab* is mapped to the hybrid entanglement between the photon *b* and the ensemble *E*_*A*_. In order to create the entanglement between *E*_*A*_ and *E*_*B*_, Bob just performs the same operations as Alice does. In brief, before and after the CPF operation on the photon *b* and the ensemble *E*_*B*_, Bob performs two local Hadamard operations on the photon *b* with *H*. These operations result in the entanglement between the photon *b* and the two atomic ensembles. The state 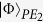 is changed into 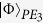



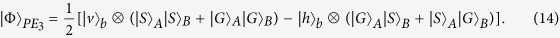


If the detector D_*h*_ at Bob’s node is clicked, the state of the system composed of *E*_*A*_ and *E*_*B*_ will be collapsed into the desired entangled state


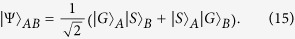


As for the case that the photon *b* is in the state 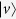, they can also obtain the desired entangled state 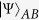 with an additional bit-flip operation 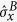 on *E*_*B*_.

### Entanglement swapping on atomic ensembles with a PCG

After the parties produce successfully the entanglement between each two atomic ensembles in the neighboring nodes, they can extend the entanglement to a further distance by entanglement swapping. Let us use the case with three nodes as an example to describe the principle for connecting the two non-neighboring nodes.

**Figure 6:**
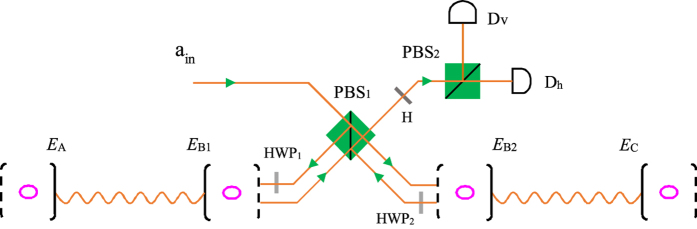
Schematic setup for entanglement swapping with the simplified PCG.

Suppose the atomic ensembles *E*_*A*_ and *E*_*C*_ belong to the two non-neighboring nodes Alice and Charlie, respectively, and the two ensembles 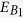 and 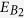 belong to the middle node Bob, shown in Fig. 6. The two ensembles 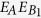 are in the state  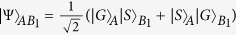 and the two ensembles 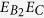 are in the state 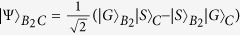. After a parity-check measurement performed on the two local ensembles 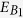 and 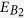 with a PCG shown in Fig. 3(b), the state of the system composed of the four ensembles *E*_*A*_, *E*_*C*_, 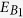, and 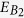 evolves into an entangled one. If the outcome of the parity-check measurement on the ensembles *B*_1_*B*_2_ is odd, the composite system composed of 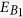, 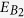, *E*_*A*_, and 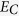 will be projected into the state


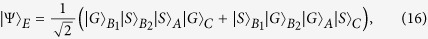


which is a four-qubit Greenberger-Horne-Zeilinger state. The decoherence of both 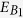 and 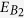 has an awful influence on the system composed of *E*_*A*_ and *E*_*C*_ as it decreases the fidelity of the entanglement of the system. In order to disentangle the two ensembles 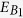 and 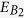 from the system, the party at the middle node could first perform a Hadamard operation on the two ensembles and then apply a parity-check measurement on them. If the outcome of the second parity-check measurement is even, the composite system composed of the four ensembles 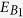, 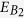, *E*_*A*_, and *E*_*C*_ is projected into the state


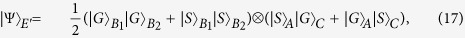


where the ensembles 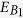 and 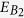 are decoupled from the system composed of the two nonlocal ensembles *E*_*A*_ and *E*_*C*_ which are in the maximally entangled state 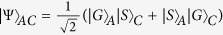.

In the discussion above, we use the outcomes (odd, even) of the two successive parity-check measurements as an example to describe the principle of the entanglement swapping between the four atomic ensembles. In fact, the other cases that the outcomes of each parity-check measurement is either an odd one or an even one can also be used for the entanglement swapping with only a single-qubit operation on the ensemble *E*_*A*_, shown in Table 1.

## Discussion

We would like to briefly discuss the imperfections of our quantum repeater protocol. The photon loss is the main imperfection, which is also of crucial importance for the previous quantum repeaters with photon interference^8^,^9^,^10^,^11^,^12^,^13^,^14^,^15^,^17^,^18^,^19^,^20^,^21^,^22^,^23^,^24^,^25^. The photon loss happens, due to the fiber absorbtion, diffraction, the cavity imperfection, and the inefficiency of the single-photon detectors. It will decrease the success probability and prolong the time needed for establishing the quantum repeater. Since the memory node in this protocol is implemented with the atomic ensemble, the local operation between two collective quantum states 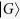 and 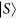 of the memory node, can be performed with collective laser manipulations^35^, while excitations of higher-order collective states can be suppressed efficiently with the Rydberg blockade^38^. During the entanglement swapping process, to detect the collective state of two ensembles in the centering nodes, fluorescent detection^44^ can be used, since the detection efficiencies of 99.99% for trapped ions have been experimentally demonstrated^45^. Moreover, with the current significant progress on the source of entangled photon pairs, the repetition rate as high as 10^6^/10^7^ *S*^−1^ has been achieved^46^, so our entanglement distribution process can be performed with a high efficiency.

In summary, we have proposed a high-efficiency quantum repeater with atomic ensembles embedded in optical cavities as the memory nodes, assisted by single-photon faithful transmission. By encoding the polarization qubit into the time-bin qubit, our faithful single-photon transmission can be completed with only linear-optical elements, and neither time-slot discriminator nor fast PCs is required^29^,^30^,^31^,^32^,^33^. The heralded entanglement creation between the neighboring nodes is achieved with a CPF gate between the atomic ensemble and the photon input in each node, which makes our scheme more convenient than the one with post selection^35^, although both efficiencies of our quantum repeaters are identical and maximal among all the exciting quantum repeater schemes when multi-mode speed up is not considered^18^,^20^. Besides, no additional classical information is involved to determinate the state of the entangled atomic ensembles, since the parties can create a deterministic entanglement up to a feedback upon the results of photon detection. The quantum swapping process is deterministically completed with a simplified PCG involving only one input-output process, which makes our scheme far more efficient than the ones based on linear optical elements^15^.

## Methods

### Faithful single-photon transmission

Our protocol for deterministic polarization-error-free single-photon transmission can be details as follows. Assuming the initial state of the single photon to be transmitted is 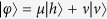 (|*μ*|^2^ + |*ν*|^2^ = 1). After passing through the encoder, the photon launched into the noisy channel evolves into


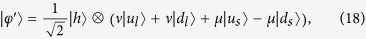


where the subscripts *l* and *s* represent the photons passing through the long path and short path of the encoder, respectively. When the optical path difference between *l* and *s* is small, the two time bins are so close that they suffer from the same fluctuation from the optical fiber channels^3^,^6^,^29^,^30^,^31^,^32^,^33^,^47^,^48^,^49^,^50^,^51^,^52^. The noise of the channel can be expressed with a unitary transformation U_*C*_ as follows:


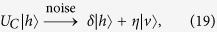


where |*δ*|^2^ + |*η*|^2^ = 1. After the photon passes through the channels, a *π* phase shifter *P*_*π*_ on the *d* channel is applied, and the state of the photon becomes


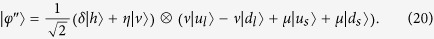


With a decoder composed of a BS, an HWP, and a PBS, shown in Fig. 5(b), the evolution of the photon can be described as follows:


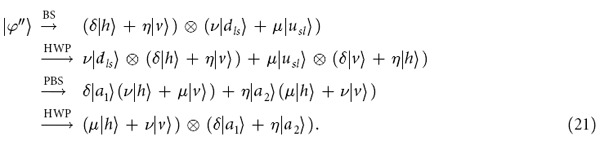


Here the subscripts *ls* (*sl*) represent the photon that passes through the long (short) path of the encoder and the short (long) path of the decoder, respectively. The difference between the long path and the short one for the encoder is designed to be the same as that for the decoder. Without any time-slot discriminator, one can get the error-free photon in either the output *a*_1_ or *a*_2_ at a deterministic time slot.

### Performance of CPF and PCG with current experimental parameters

Before we analyze the fidelity of the quantum entanglement distribution and entanglement swapping in our quantum repeater scheme, we first discuss the practical performance of the CPF gate and the PCG based on the recent experiment advances^53^,^54^,^55^. We define the fidelity of a quantum process (or a quantum gate) as 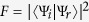, where 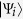 and 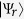 are the output states of the quantum system in the quantum process (or the quantum gate) in the ideal condition and the realistic condition, respectively^15^.

By combining a fibre-based cavity with the atom-chip technology, Colombe *et al.*^53^ demonstrated the strong atom-field coupling in a recent experiment in which each ^87^Rb atom in Bose-Einstein condensates is identically and strongly coupled to the cavity mode. In this experiment, all the atoms are initialized to be the hyperfine zeeman state 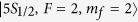. The dipole transition of ^87^Rb 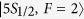 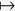 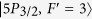 is resonantly coupled to the cavity mode with the maximal single-atom coupling strength *g*_0_ = 2*π* × 215 MHz. Meanwhile, the cavity photon decay rate is *κ* = 2*π* × 53 MHz and the atomic spontaneous emission rate of 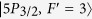 is *γ* = 2*π* × 3 MHz. The whispering-gallery microcavities (WGMC)^56^ might be another potential experimental realization of our scheme. The parity-time-symmetry breaking is realized in a system of two directly coupled WGMC^57^ and the controlled loss is also achieved with WGMC^58^, which enables the on-chip manipulation and control of light propagation. In addition, the routing of single photons has been demonstrated by the atom-WGMC coupled unit controlled by a single photon^59^.

Under an ideal condition, the reflection coefficients of the input-output processes are 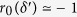 and 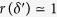. In this time, the input 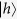 polarized photon *a* will get a *π* phase shift when the embedded atomic ensemble *E*_*A*_ is in the state 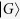; otherwise, there is no phase shift on the photon *a*. The fidelity of both the CPF gate (shown in Fig. 4(a)) and the PCG (shown in Fig. 4(b)) can reach unity. In a realistic atom-cavity system, the relationship between the input and output field is outlined in Eqs (3) and (4). In this time, after the party operates the photon *a* and the ensemble *E*_*A*_ with the CPF gate, the output state of the composite system becomes


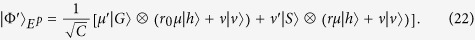


Here the normalized coefficient *C* = |*r*_0_ ⋅ *μ*′ ⋅ *μ*|^2^ + |*r* ⋅ *ν*′ ⋅ *μ*|^2^ + |*μ*′ ⋅ *ν*|^2^ + |*ν*′ ⋅ *ν*|^2^. The fidelity of the CPF gate 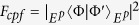 depends on the input state of the system composed of the photon and the atomic ensemble. In the symmetric case with 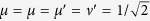, the fidelity *F*_*cpf*_ can be simplified to be


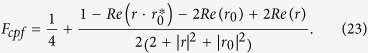


Meanwhile, the efficiency *η*_*cpf*_ of the CPF gate, which is defined as the probability that the photon clicks either detectors after being reflected by the CPF gate, can be detailed as


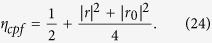


In a realistic condition, the output state of the composite system composed of *a*, *E*_*A*_, and *E*_*B*_ in the PCG process before the single photon is detected becomes


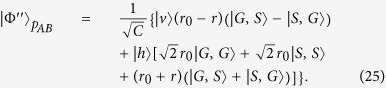


Compared with the ideal output state described in Eq. (7), if an 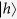 polarized photon is detected, the fidelity of the PCG gate *F*_*pcg*_ can be expressed as


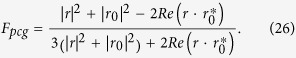


When the photon in the state 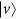 is detected, the fidelity of the PCG is 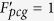. The success of the PCG is heralded when a single photon is detected after the parity-check process, no matter what the state the photon evolves to be. The efficiency *η*_*pcg*_ of the PCG process can be defined as the probability that the probe photon is detected after it is reflected by the two cavities, that is,


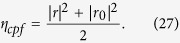


Since the absolute value of the relative phase shift during the input-output process depends on the frequency of the input photon, it decreases smoothly with the detuning *δ*′ between the input photon and the cavity mode, shown in Fig. 3.

**Figure 7:**
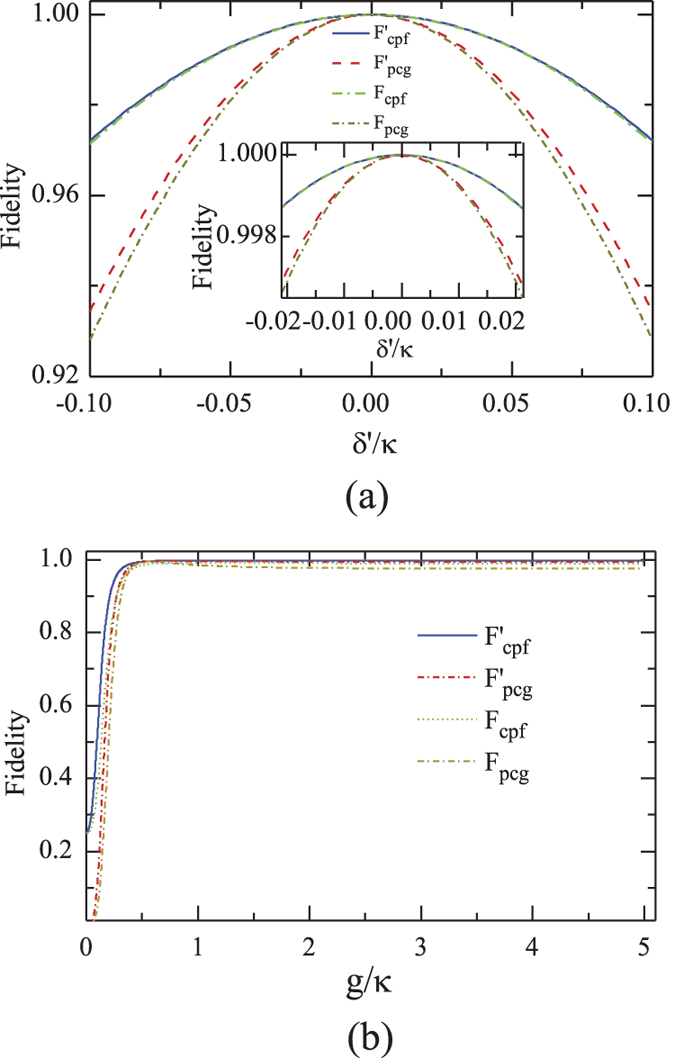
(**a**) Fidelities of our CPF gate and PCG *vs* the scaled detuning. 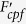 and 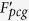 is performed with the scaled coupling rate *g*/*κ* = 2.0283 and *γ*/*κ* = 0.0566, *F*_*cpf*_ and *F*_*pcg*_ are performed with the scaled coupling rate *g*/*κ* = 4.0566 and *γ*/*κ* = 0.0566^53^. (**b**) Fidelities of our CPF gate and PCG gate *vs* the scaled coupling rate. 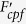 and 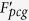 are performed with the scaled detuning *δ*′/*κ* = 0.0283 and *γ*/*κ* = 0.0566, *F*_*cpf*_ and *F*_*pcg*_ is performed with *δ*′/*κ* = *γ*/*κ* = 0.0566.

The fidelity of the CPF gate *F*_*cpf*_ changes with the detuning *δ*′, shown in Fig. 7(a). Here the parameters are chosen as *g*/*κ* = 2.0283 or 4.0566 and *γ*/*κ* = 0.0566^53^. When the linewidth of the input photon is *δ* = 2|*δ*′|_*max*_ with the maximal detuning |*δ*′|_*max*_ = 0.5*γ* (*γ*), *F*_*cpf*_ is larger than *F*_*cpf*_(|*δ*′|_*max*_) = 0.9974 (0.9906) for *g*/*κ* = 4.0566.

The fidelity of the PCG depends on the coupling rate *g*/*κ*, as shown in Fig. 7(b) with the detuning |*δ*′|_*max*_ = 0.5 *γ* or *γ*. When the maximal detuning of the input photon is |*δ*′|_*max*_ = 0.5 *γ*, the high-performance parity-check gate can be achieved with the fidelity *F*_*pcg*_ higher than *F*_*pcg*_(|*δ*′|_*max*_) = 0.9944 and 0.9938 for *g*/*κ* = 2.0283 and *g*/*κ* = 4.0566, respectively.

**Figure 8:**
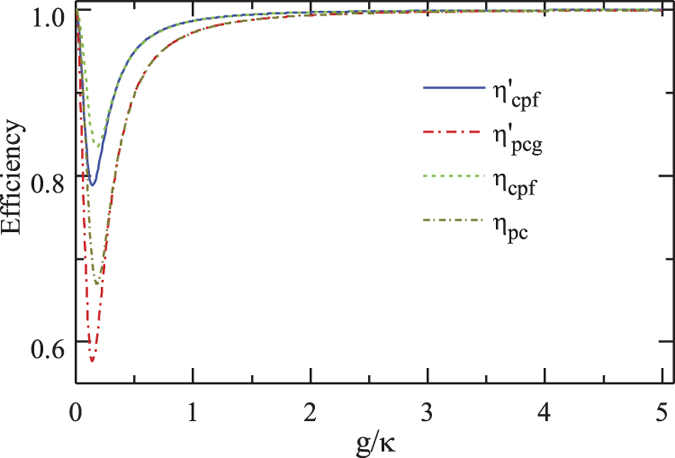
Efficiencies of our CPF gate and PCG *vs* the scaled coupling rate. 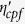
 and 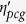 is performed with the scaled detuning *δ*′/*κ* = 0.0283 and *γ*/*κ* = 0.0566, *η*_*cpf*_ and *η*_*pcg*_ are performed with *δ*′/*κ* = *γ*/*κ* = 0.0566.

The efficiencies of the CPF gate and the PCG process versus the coupling rate *g*/*κ* are shown in Fig. 8. When the bandwidth of the probe photon is on the scale of *γ*, both efficiencies *η*_*cpf*_ and *η*_*pcg*_ are robust to the variation of *g*/*κ* with the parameters above^53^. In detail, when the maximal detuning |*δ*′|_*max*_ of the input photon is less than 0.5 *γ*, *η*_*cpf*_ and *η*_*pcg*_ are higher than 0.9966 and 0.9932, respectively. When |*δ*′|_*max*_ = *γ*, *η*_*cpf*_ = 0.9991 and *η*_*pcg*_ = 0.9983 are achievable.

### Performance of entanglement distribution and entanglement swapping

Now, let us discuss the fidelities and the efficiencies of the entanglement distribution and entanglement swapping in our quantum repeater scheme. After Alice performs the local operations on the photon *a* and detects an 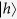 polarized photon, the composite system composed of the photon *b* and the ensembles *E*_*A*_ and *E*_*B*_ will be projected into the state 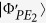, instead of 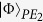. Here


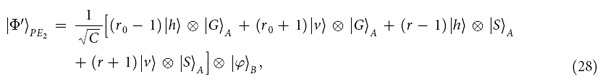


where the normalized coefficient *C* = 2[|*r*_0_ − 1|^2^ + |*r*_0_ + 1|^2^ + |*r* − 1|^2^ + |*r* + 1|^2^]. And then, the same operations, i.e., a CPF gate sandwiched by two Hadamard operations, are performed by Bob on the photon *b*. After these operations, the state of the composite system composed of the photon *b* and the two ensembles *E*_*A*_ and *E*_*B*_ evolves into


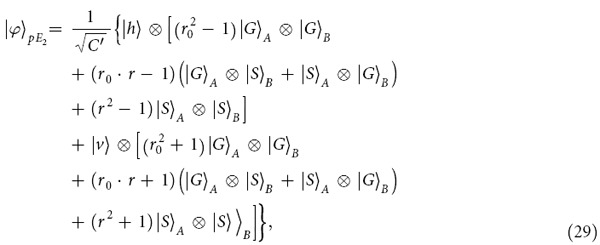


where the normalized coefficient 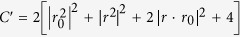. One can obtain the fidelity of the entanglement distribution process *F*_*mh*_ and *F*_*mv*_ for the cases that *D*′_*h*_ and *D*′_*v*_ at the Bob’s node are clicked, respectively.


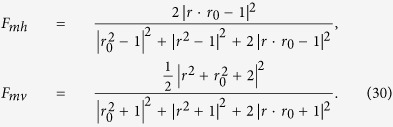


If one defines the efficiency 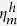 as the probability that Alice detects an 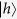 polarized photon while Bob detects a photon in either 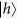 or 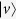 polarization, one has


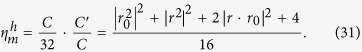


In the above discussion, we detail the performance of our entanglement distribution conditioned on the detection of an 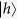 polarization photon at Alice’s node. Considering the symmetric property of the system, one can easily obtain the performance of the entanglement distribution upon the detection of a 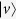 polarization photon at Alice’s node. Now, the fidelities 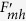 and 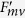 for the cases that *D*′_*h*_ and *D*′_*v*_ are clicked at Bob’s node, have the following relations to that for the cases that an 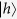 polarized photon is detected by Alice, 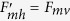 and 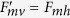, see Eq. (30) for detail. Meanwhile, the efficiency 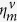 of the entanglement distribution process when Alice detects a photon in 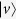 polarization is identical to 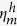. The total efficiency *η*_*m*_ of the entanglement distribution can be written as


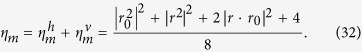


In our entanglement swapping process, two PCGs are applied on the two ensembles 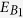 and 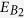 at the middle node. In fact, only one PCG is enough if a single-atomic-ensemble measurement on each of the two ensembles *E*_*B*1_ and *E*_*B*2_ is utilized after the local Hadamard operations. After these measurements, the system composed of the two remote ensembles *E*_*A*_ and *E*_*C*_ is in the state 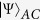 with or without a local unitary operation. When the fluorescent measurement^44^ or field-ionizing the atoms^28^ with the help of Rydberg excitation are used, the state detection on atomic ensembles could be performed with a near-unity efficiency. In other words, the fidelity of the quantum entanglement swapping process can equal to that of the PCG operation.

**Figure 9:**
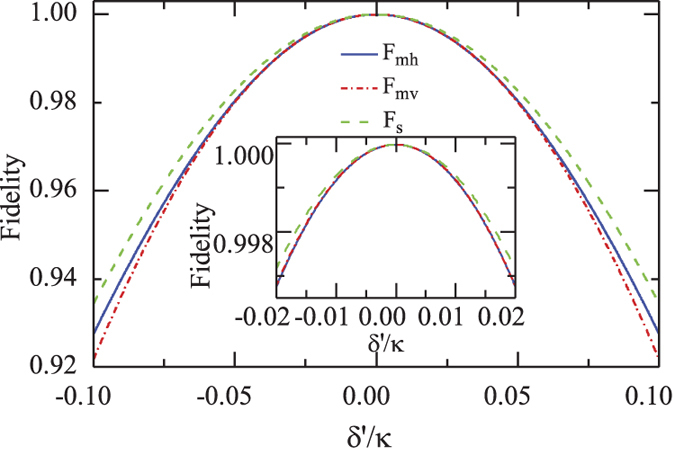
Fidelities of F_*mh*_, F_*mv*_ and F_*s*_ vs the detuing, with the scaled coupling rate *g*/*κ* = 2.0283 and *γ*/*κ* = 0.0566.

**Figure 10:**
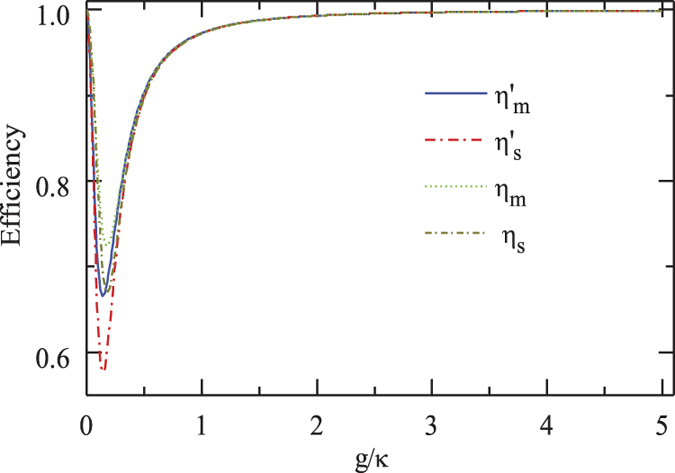
Efficiencies of entanglement distribution and entanglement swapping processes *vs* the scaled coupling rate. 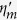
 and 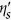 is performed with the scaled detuning *δ*′/*κ* = 0.0283 and *γ*/*κ* = 0.0566, *η*_*m*_ and *η*_*s*_ are performed with *δ*′/*κ* = *γ*/*κ* = 0.0566.

The fidelities of both the entanglement distribution and the entanglement swapping in our repeater scheme are shown in Fig. 9. One can see that all *F*_*mh*_, *F*_*mv*_, and *F*_*s*_ = *F*_*pcg*_ are larger than 0.9936 with the parameters (*g*, *κ*, *γ*) = 2*π* × (215, 53, 3) MHz achieved in experiment^53^. Meanwhile, all efficiencies involved in our quantum repeater protocol, shown in Fig. 10, can be larger than 0.9931 when the effective coupling *g*/*κ* > 2.0283 with *δ*′/*κ* = *γ*/*κ* = 0.0566. In a recent experiment with a fiber-based Fabry*-*Perot cavity constituted by CO_2_ laser-machined mirrors^60^, the maximal coupling strength as high as *g* = 2*π* × 2.8 GHz is achieved for single Rb atoms and the cavity decay rate is *κ* = 2*π* × 0.286 GHz ≃95*γ*. In this time, *g*/*κ* = 9.79 is achieved, and a better performance of our scheme is attainable.

## Additional Information

**How to cite this article**: Li, T. and Deng, F.-G. Heralded high-efficiency quantum repeater with atomic ensembles assisted by faithful single-photon transmission. *Sci. Rep.* **5**, 15610; doi: 10.1038/srep15610 (2015).

## Figures and Tables

**Table 1:** The relation between the single-qbuit operation on the ensemble *E*_*A*_ for entanglement swapping and the outcomes of the parity-check measurements on the two atomic ensembles at the middle node.

P_1_	P_2_	E_*A*_
*v*	*h*	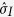
*v*	*v*	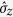
*h*	*v*	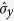
*h*	*h*	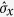

*P*_1_ and *P*_2_ denote the outcomes of the first and the second parity-check measurements. Here 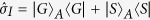, 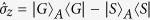, 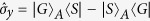, and 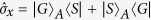.
